# Tea and coffee and risk of endometrial cancer: cohort study and meta-analysis^1^,^2^,^3^,^4^

**DOI:** 10.3945/ajcn.113.081836

**Published:** 2015-01-21

**Authors:** TienYu Owen Yang, Francesca Crowe, Benjamin J Cairns, Gillian K Reeves, Valerie Beral

**Affiliations:** 1 From the Nuffield Department of Population Health, University of Oxford, Oxford, United Kingdom.

**Keywords:** caffeine, coffee, endometrial cancer, tea, meta-analysis

## Abstract

**Background:** Previous reports, mostly from retrospective studies, suggested possible protective effects of both tea and coffee against endometrial cancer, but recent reports from prospective studies generally showed weaker or null associations.

**Objectives:** We investigated endometrial cancer risk in relation to tea and coffee consumption in a large prospective study and did a meta-analysis of published results.

**Design:** Daily consumption of tea and coffee was recorded in 560,356 participants (without a hysterectomy) in the UK Million Women Study of whom 4067 women developed endometrial cancer during 5.2 million person-years of follow up (average: 9.3 y per woman).

**Results:** With the use of Cox proportional hazards regression, we showed no significant association between endometrial cancer risk and consumption of either tea (multivariate adjusted RR per cup daily: 1.00; 95% CI: 0.98, 1.02) or coffee (RR per cup daily: 0.98; 95% CI: 0.96, 1.01). Our meta-analyses showed no significant association between endometrial cancer risk and tea consumption and a weak association for coffee consumption in prospective studies, but there may have been selective publication of only part of the evidence.

**Conclusions:** There is little or no association between tea consumption and endometrial cancer risk. If there is any association with coffee consumption, it appears to be weak.

## INTRODUCTION

Previous studies suggested that consumption of tea (1) and coffee (2, 3) may be associated with a reduction in risk of endometrial cancer. Results of the most-recent meta-analysis of tea consumption (1) were influenced by results from retrospective studies, which were potentially biased by differential recall between cases and control subjects. For coffee consumption, one meta-analysis (3) reported that associations were weaker for prospective than retrospective studies. We investigated risk of endometrial cancer in relation to tea and coffee consumption in a large prospective study of United Kingdom women, a population of whom are known to have relatively high consumption of these beverages. We also report updated meta-analyses of these associations.

## METHODS

### Study and follow-up

In the Million Women Study (4), ∼1.3 million middle-aged women were recruited when they were invited to a routine breast cancer screening in England and Scotland between 1996 and 2001. Participants gave informed consent and returned a recruitment questionnaire on health, lifestyle, and reproductive factors at their breast-screening appointment. Approximately 3 y after recruitment, 65% of participants responded to the invitation to complete a resurvey that provided additional and updated information, including details of their diet and beverage consumption. Full study questionnaires are shown on the Million Women Study website (www.millionwomenstudy.org). All participants were flagged in the National Health Service Central Registers, which routinely provide investigators with information on deaths and incident cancers, and coded according to the International Classification of Diseases, 10th Revision. The study was approved by the Oxford and Anglia Multi-Centre Research and Ethics Committee.

### Exposure

Women were asked to report the numbers of cups of tea and coffee they consumed each day during a typical week. Women were also asked to report whether they added milk to their tea or coffee and at what frequency (none, sometimes, usually, or always). For these analyses, tea and coffee consumption was divided into 4 categories of <1, 1–2, 3–4, and ≥5 cups/d. The short-term and long-term stability of self-reported tea or coffee consumption in this cohort was already reported (5). Mean self-reported consumption of tea and coffee was stable in the shorter term (around 6 mo) and longer term (around 2 y).

### Statistical analysis

The analysis was restricted to women who provided information on consumption of tea, coffee, water, and soft drinks and had no cancer registered before the study baseline. Women were excluded if they reported having had or being unsure if they had had a hysterectomy. Because BMI (in kg/m^2^) is a known strong risk factor for endometrial cancer (6, 7), all analyses were further restricted to women who provided information on height and weight. Participants were followed from the date when they reported tea and coffee consumption (study baseline; at mean ± SD age: 58.8 ± 4.6 y) to the date of any cancer registration (except nonmelanoma skin cancer); date of death, emigration, or other loss to follow-up; or last date of follow-up (31 December 2011), whichever was earliest.

In all analyses, the endpoint of interest was endometrial cancer (International Classification of Diseases, 10th Revision code C54). RRs of cancer by tea or coffee consumption were estimated by using Cox proportional hazards regression with the attained age as the underlying time variable. In analyses by categories of tea or coffee consumption, we used 1–2 cups/d as the reference group, and 95% group-specific CIs (8) were presented to allow comparison of risks between any 2 groups, even when neither was the reference group. Four years, on average, after baseline, 57% of women provided repeat responses to the same questions about tea and coffee consumption, and we examined the repeatability of these questions by using Spearman’s correlation between 2 measures. RRs of endometrial cancer per additional cup of tea or coffee daily (cups/d) were estimated as the trend across the 4 categories for each exposure. To correct for regression dilution because of measurement error, each category was assigned mean consumption from repeat responses to the tea or coffee question from women in that category at baseline (9). The validity of the assumption of proportional hazards was tested via Schoenfeld’s residuals by using the estat phtest command in STATA 13.1 software (StataCorp LP).

All analyses were stratified by the region of residence (Scotland and 9 cancer-registration regions in England) and socioeconomic status [Townsend deprivation index (10) in fifths], and adjusted for adult height (<155, 155–159, 160–164, 165–169, 170–174, and ≥175 cm), age at menarche (<12, 12, 13, 14, and ≥15 y old), parity (nulliparous, 1–2 children, and ≥3 children), use of oral contraceptives (ever or never), age and status of menopause (premenopause; ≤46, 47–49, 50, 51–53, and ≥54 y old; and not sure), use of hormone therapy for menopause (never, former user having stopped <5 or ≥5 y, current user, and ever user with unknown current status of use), BMI (<20, 20.0–22.4, 22.5–24.9, 25.0–27.4, 27.5–29.9, 30.0–32.4, 32.5–34.9, and ≥35.0), smoking status (never, former smoker having quit <10 or ≥10 y, and current smoker of <15, 15–24, or ≥25 cigarettes/d), alcohol consumption (0, 1–4, 5–14, 15–29, and 30 g/d) (11), strenuous exercise per week (never, less than once, and once or more), coffee or tea consumption (as appropriate; 0, 1–2, 3–4, and ≥5 cups/d), and other nonalcohol fluid intake (sum of daily intake of milk, squash, water, fizzy drinks, and juice in fifths). The small number of subjects with missing values of these adjustment variables (<2.5% for each) were assigned to a separate category.

### Meta-analysis

A meta-analysis was conducted to estimate risk of endometrial cancer per additional cup of tea or coffee daily in published studies and reported according to the Meta-analysis of Observational Studies in Epidemiology statement (12). All studies with published risks of endometrial cancer associated with tea or coffee consumption were included. No limitation was applied in terms of study size, study population, date, or language of publication. Two independent researchers (TOY and FC) searched in PubMed (http://www.ncbi.nlm.nih.gov/pubmed) and Embase (http://www.elsevier.com/online-tools/embase) by using combinations of keywords (coffee OR caffein* OR tea OR flavonoid OR catechin OR thearubigin OR theaflavin) AND (endometri* cancer OR endometri* neoplas* OR uterine cancer OR uterine neoplas*) and also searched reference lists of articles identified from the search. The researchers independently reviewed the full articles and extracted information from each study on RRs of endometrial cancers as well as the type of tea consumed wherever specified (e.g., black tea or green tea), and mean consumption of tea or coffee.

In studies that gave only RRs of endometrial cancer across different categorical levels of consumption, trends were estimated by using generalized least-squares method with the glst package in STATA 13.1 software (13). In most studies, median or mean values of each category of consumption were not available for a trend estimation, and thus, the midpoint of each category was used, and for the top (open) category, the lower bound plus the width of the second-highest category was used (14). We used inverse-variance weighted meta-analysis to combine the effects of endometrial cancer risk per additional cup of tea or coffee daily, adjusted to 200 mL or 2 g of dry tea each cup as appropriate, with the STATA command metan (15). Heterogeneity was assessed with *P* values by using chi-square tests.

## RESULTS

Of 560,356 women followed for an average of 9.3 y, 4067 cases of endometrial cancer were registered. Mean (±SD) consumption reported at baseline was 3.4 ± 2.7 cups tea/d and 2.0 ± 2.0 cups coffee/d. **Table 1** shows Spearman’s correlation coefficients between reports of consumption at baseline and later (0.78 for tea consumption and 0.78 for coffee consumption measured 2 y after baseline with slightly more attenuation after longer periods).

**TABLE 1:** Repeatability of self-reported daily tea or coffee consumption in the Million Women Study by duration between 2 self-reports

	Tea		Coffee	
Time between 2 reports	*n*	Spearman’s correlation	*n*	Spearman’s correlation
0–2 y	27,514	0.78	27,514	0.78
3–5 y	271,188	0.74	271,188	0.71
≥6 y	23,083	0.71	23,083	0.67

For tea consumption, women with the highest consumption (≥5 cups/d) and lowest consumption (less than daily) were more likely than other tea drinkers to be in the lowest fifth of socioeconomic status, be current smokers, and not engage in strenuous physical activity (**Table 2**). These associations were similar for coffee consumption except that women who consumed the most coffee (≥5 cups/d) were much-more likely to be current smokers. Women who consumed the most tea or coffee tended to consume less water and other soft drinks.

**TABLE 2:** Characteristics according to daily consumption of tea or coffee

	Daily tea consumption at study baseline (cups/d)				Daily coffee consumption at study baseline (cups/d)				
	<1 (*n* = 87,124)	1–2 (*n* = 130,682)	3–4 (*n* = 184,707)	≥5 (*n* = 157,843)	<1 (*n* = 140,498)	1–2 (*n* = 246,885)	3–4 (*n* = 118,551)	≥5 (*n* = 54,422)	All women (*n* = 560,356)
Tea consumption repeated 4 y later, cups/d	0.8 ± 2.1^1^	2.4 ± 2.3	4.0 ± 3.0	6.1 ± 4.2	—	—	—	—	3.9 ± 3.6
Coffee consumption repeated 4 y later, cups/d	—	—	—	—	0.6 ± 1.3	1.7 ± 1.7	3.3 ± 2.8	5.3 ± 4.2	2.3 ± 2.7
Age at baseline, y	59 ± 5	59 ± 5	60 ± 5	60 ± 5	59 ± 5	60 ± 5	59 ± 5	58 ± 5	59 ± 5
Age at menarche, y	13 ± 2	13 ± 2	13 ± 2	13 ± 2	13 ± 2	13 ± 2	13 ± 2	13 ± 2	13 ± 2
Adult height, cm	162 ± 7	163 ± 7	162 ± 7	162 ± 7	162 ± 7	162 ± 7	163 ± 7	162 ± 7	162 ± 7
Nulliparity, %	13	13	11	11	12	12	12	12	12
Oral contraceptive use, %	66	65	60	57	60	60	64	66	61
Hormone therapy for menopause, %	48	49	48	46	49	47	47	46	48
Lowest fifth of socioeconomic status, %	21	17	19	23	25	18	17	22	20
BMI, kg/m^2^	27 ± 5	26 ± 4	26 ± 4	26 ± 5	26 ± 5	26 ± 4	26 ± 5	27 ± 5	26 ± 5
Current smoker, %	16	10	9	15	11	9	14	26	12
Strenuous exercise <1 time/wk, %	58	54	56	60	60	55	56	61	57
Alcohol consumption, g/d	7 ± 10	8 ± 9	7 ± 8	5 ± 8	5 ± 8	7 ± 9	8 ± 9	7 ± 9	7 ± 9
Consumption of other nonalcoholic fluid, glasses/d	5 ± 8	5 ± 5	4 ± 5	4 ± 6	5 ± 7	4 ± 6	4 ± 5	4 ± 5	4 ± 6

1 Mean ± SD (all such values).

There were no significant differences in endometrial cancer risk across 4 categories of tea consumption after adjustment for region and socioeconomic status only (<1, 1–2, 3–4, and ≥5 cups/d; *P*-heterogeneity = 0.2; **Table 3**). The assumption of proportional hazards was not violated (*P* > 0.05). There was no increased or decreased risk of endometrial cancer per additional cup of tea daily (RR: 1.00; 95% CI: 0.98, 1.01), and the estimate did not change after multivariate adjustment (RR: 1.00; 95% CI: 0.98, 1.02). The association remained unchanged when we restricted the analysis to daily drinkers (multivariately adjusted RR: 1.00; 95% CI: 0.97, 1.02). There was no significant heterogeneity in trends across subgroups by smoking, BMI, or whether milk was added to tea.

**TABLE 3:** RRs (95% CIs or group-specific 95% CIs) for associations of endometrial cancer risk with tea consumption^1^

	Tea consumption (cups/d)					Trend (/cup)		
	<1	1–2	3–4	≥5	*P*-heterogeneity	All	Daily consumers only	*P*-heterogeneity across subgroups
All								
Cases	647	893	1374	1153	—	4067	3420	
RR^2^ (95% CI)	1.11 (1.02, 1.20)	1.00 (0.94, 1.07)	1.07 (1.01, 1.13)	1.04 (0.98, 1.11)	0.2	1.00 (0.98, 1.01)	1.01 (0.98, 1.03)	
RR^3^ (95% CI)	1.04 (0.96, 1.14)	1.00 (0.94, 1.07)	1.05 (1.00, 1.11)	1.01 (0.95, 1.08)	0.6	1.00 (0.98, 1.02)	1.00 (0.97, 1.02)	
Smoking								
Never smoked								
Cases	344	539	850	674	—	2407	—	
RR^3^ (95% CI)	0.98 (0.87, 1.10)	1.00 (0.92, 1.09)	1.03 (0.96, 1.10)	0.96 (0.88, 1.04)	0.6	0.99 (0.97, 1.02)	—	
Former smoker								
Cases	222	285	406	346	—	1259	—	
RR^3^ (95% CI)	1.14 (0.98, 1.32)	1.00 (0.89, 1.12)	1.04 (0.95, 1.15)	1.07 (0.95, 1.20)	0.6	1.00 (0.96, 1.03)	—	
Current smoker								
Cases	66	57	83	102	—	308	—	
RR^3^ (95% CI)	1.04 (0.78, 1.38)	1.00 (0.77, 1.30)	1.22 (0.98, 1.51)	1.10 (0.86, 1.41)	0.8	1.02 (0.95, 1.09)	—	0.8
BMI (kg/m^2^)								
<25.0								
Cases	155	283	428	305	—	1171	—	
RR^3^ (95% CI)	0.94 (0.79, 1.12)	1.00 (0.89, 1.12)	1.11 (1.00, 1.22)	0.99 (0.87, 1.12)	0.3	1.01 (0.97, 1.04)	—	
25.0–29.9								
Cases	215	310	514	436	—	1475	—	
RR^3^ (95% CI)	1.10 (0.95, 1.28)	1.00 (0.89, 1.12)	1.07 (0.98, 1.17)	1.04 (0.93, 1.16)	0.7	1.00 (0.97, 1.03)	—	
30.0–34.9								
Cases	133	174	256	233	—	796	—	
RR^3^ (95% CI)	1.00 (0.83, 1.22)	1.00 (0.86, 1.16)	0.95 (0.84, 1.07)	0.91 (0.78, 1.05)	0.8	0.98 (0.94, 1.02)	—	
≥35.0								
Cases	143	126	175	177	—	621	—	
RR^3^ (95% CI)	1.15 (0.95, 1.39)	1.00 (0.84, 1.19)	1.04 (0.89, 1.20)	1.12 (0.95, 1.33)	0.6	1.00 (0.96, 1.05)	—	0.8
Adding milk to tea								
Never								
Cases	220	116	112	67	—	515	—	
RR^3^ (95% CI)	0.97 (0.83, 1.14)	1.00 (0.83, 1.20)	1.02 (0.85, 1.23)	0.82 (0.64, 1.06)	0.35	0.98 (0.93, 1.03)	—	
Sometimes or usually								
Cases	58	174	186	125	—	543	—	
RR^3^ (95% CI)	1.04 (0.79, 1.37)	1.00 (0.86, 1.16)	0.96 (0.83, 1.11)	1.00 (0.83, 1.21)	0.95	1.00 (0.94, 1.05)	—	
Always								
Cases	146	594	1066	957	—	2763	—	
RR^3^ (95% CI)	1.07 (0.90, 1.26)	1.00 (0.92, 1.08)	1.08 (1.01, 1.15)	1.03 (0.96, 1.11)	0.5	1.00 (0.98, 1.03)	—	0.7

1 RRs were estimated by using Cox regression. Group-specific 95% CIs were used to allow comparison of risks between any 2 groups even when neither was the reference group.

2 RR with age as an underlying variable. Risks were adjusted for region and socioeconomic status.

3 RR with age as an underlying variable. Risks were adjusted for region, socioeconomic status, height, age at menarche, parity, duration of oral contraceptive use, age and status of menopause at study baseline, duration of hormone therapy for menopause, BMI, smoking, alcohol consumption, strenuous exercise, coffee consumption, and other nonalcoholic fluid intake.

There were also no significant differences in endometrial cancer risk across 4 categories of coffee consumption (0, 1–2, 3–4, and ≥5 cups/d, *P*-heterogeneity = 0.8; **Table 4**). With adjustment for region and socioeconomic status, the RR of endometrial cancer per additional cup of coffee daily was 0.99 (95% CI: 0.97, 1.01), and the estimate did not change substantially after multivariate adjustment (RR: 0.98; 95% CI: 0.96, 1.01). The proportional hazards assumption was not violated (*P* > 0.05). The RR was not altered when we restricted the analysis to daily drinkers (multivariate-adjusted RR: 0.97; 95% CI: 0.94, 1.01). There was some evidence of heterogeneity (*P* = 0.007) between trends of 4 subgroups by BMI. The multivariate-adjusted RR per additional cup of coffee daily in women with BMI <25.0 was 1.04 (95% CI: 0.99, 1.09) compared with an RR of 0.98 (95% CI: 0.94, 1.02) in women with BMI of 25.0–29.9 kg/m^2^, 0.91 (95% CI: 0.86–0.97) in women with a BMI of 30.0–34.9, and 0.99 (95% CI: 0.93–1.05) in women with a BMI ≥35.0. There was little to suggest heterogeneity in RRs across subgroups by smoking status (*P* = 0.2) or whether milk was added to coffee (*P* = 0.5).

**TABLE 4:** RRs (and 95% CIs or group-specific 95% CIs) for associations of endometrial cancer risk with coffee consumption^1^

	Coffee consumption (cups/d)					Trend (/cup)		
	<1	1–2	3–4	≥5	*P*-heterogeneity	All	Daily consumers only	*P*-heterogeneity across subgroups
All								
Cases	1009	1839	842	377		4067	3058	
RR^2^ (95% CI)	0.99 (0.93, 1.06)	1.00 (0.95, 1.05)	0.97 (0.90, 1.04)	0.96 (0.87, 1.06)	0.8	0.99 (0.97, 1.01)	0.99 (0.96, 1.02)	
RR^3^ (95% CI)	0.99 (0.92, 1.06)	1.00 (0.95, 1.05)	0.94 (0.88, 1.01)	0.92 (0.82, 1.03)	0.4	0.98 (0.96, 1.01)	0.97 (0.94, 1.01)	
Smoking								
Never smoked								
Cases	602	1134	500	171		2407	—	
RR^3^ (95% CI)	0.98 (0.90, 1.08)	1.00 (0.94, 1.07)	1.00 (0.91, 1.09)	0.89 (0.76, 1.04)	0.6	0.99 (0.95, 1.02)	—	
Former smoker								
Cases	310	580	250	119		1259	—	
RR^3^ (95% CI)	0.94 (0.83, 1.06)	1.00 (0.92, 1.09)	0.82 (0.73, 0.93)	0.82 (0.67, 1.00)	0.06	0.96 (0.92, 1.00)	—	
Current smoker								
Cases	65	92	76	75		308	—	
RR^3^ (95% CI)	1.06 (0.80, 1.40)	1.00 (0.81, 1.24)	1.09 (0.87, 1.37)	1.21 (0.92, 1.60)	0.8	1.04 (0.95, 1.12)	—	0.2
BMI (kg/m^2^)								
<25.0								
Cases	270	530	274	97		1171	—	
RR^3^ (95% CI)	1.00 (0.88, 1.14)	1.00 (0.91, 1.10)	1.20 (1.06, 1.35)	1.10 (0.89, 1.37)	0.1	1.04 (0.99, 1.09)	—	
25.0–29.9								
Cases	363	682	292	138		1475	—	
RR^3^ (95% CI)	1.01 (0.90, 1.13)	1.00 (0.92, 1.08)	0.90 (0.80, 1.01)	0.98 (0.81, 1.17)	0.5	0.98 (0.94, 1.02)	—	
30.0–34.9								
Cases	220	357	146	73		796	—	
RR^3^ (95% CI)	1.06 (0.91, 1.24)	1.00 (0.89, 1.12)	0.77 (0.65, 0.90)	0.74 (0.57, 0.95)	0.009	0.91 (0.86, 0.97)	—	
≥35.0								
Cases	154	270	128	69		621	—	
RR^3^ (95% CI)	0.82 (0.69, 0.99)	1.00 (0.88, 1.14)	0.87 (0.73, 1.03)	0.80 (0.62, 1.04)	0.09	0.99 (0.93, 1.05)	—	0.007
Adding milk to coffee								
Never								
Cases	183	171	130	66		550	—	
RR^3^ (95% CI)	1.04 (0.88, 1.24)	1.00 (0.86, 1.17)	1.01 (0.85, 1.20)	0.89 (0.68, 1.15)	0.78	0.97 (0.91, 1.03)	—	
Sometimes or usually								
Cases	108	351	172	62		693	—	
RR^3^ (95% CI)	1.02 (0.83, 1.24)	1.00 (0.90, 1.12)	0.91 (0.79, 1.06)	0.81 (0.62, 1.06)	0.46	0.95 (0.89, 1.01)	—	
Always								
Cases	413	1302	532	246		2493	—	
RR^3^ (95% CI)	0.95 (0.86, 1.06)	1.00 (0.94, 1.06)	0.94 (0.86, 1.02)	0.95 (0.83, 1.10)	0.6	0.99 (0.96, 1.03)	—	0.5

1 RRs were estimated by using Cox regression. Group-specific 95% CIs were used to allow comparison of risks between any 2 groups even when neither was the reference group.

2 RR with age as an underlying variable. Risks were adjusted for region and socioeconomic status.

3 RR with age as an underlying variable. Risks were adjusted for region, socioeconomic status, height, age at menarche, parity, duration of oral contraceptive use, age and status of menopause at study baseline, duration of hormone therapy for menopause, BMI, smoking, alcohol consumption, strenuous exercise, tea consumption, and other nonalcoholic fluid intake.

We investigated RRs of endometrial cancer by histology according to the International Classification of Diseases for Oncology, Morphology of Neoplasms, Second or Third Edition. There were 3386 endometrioid carcinomas (International Classification of Diseases for Oncology, Morphology of Neoplasms, Second or Third Edition codes 8380, 8382, 8383, 8480–8482, 8210, 8140, 8560, and 8570) and 171 serous carcinomas (codes 8441 and 8460–8461) registered. For tea consumption, respective RRs per additional cup daily were 1.00 (95% CI: 0.98, 1.02) and 1.03 (95% CI: 0.94, 1.13). For coffee consumption respective RRs per additional cup daily were 0.98 (95% CI: 0.95, 1.01) and 0.99 (95% CI: 0.87, 1.13).

As a sensitivity analysis, we excluded the first 4 y of follow-up, but findings were little changed (**Supplemental Table 1**). Women also reported the amount of sugar they added each day to “cereal, tea, coffee, or fruit etc.” RRs of endometrial cancer per cup of tea or coffee daily after additional adjustment for this variable (<1, 1–2, ≥3 spoons sugar added/d) were not substantially changed (Supplemental Table 1).

After the exclusion of 2 duplicate publications from the same study (16, 17) and one study in which CIs or *P* values for RRs were not available (18), the meta-analyses included 12 studies for tea consumption (18–29) and 16 studies for coffee consumption (18, 21, 22, 24, 26–37). (**Supplemental Tables 2** and **3**). All analyses were subdivided by study design (prospective and retrospective recordings of beverage consumption) and geographical region (Europe and North America as well as Asia) particularly because the type of tea consumed varies.

**FIGURE 1:**
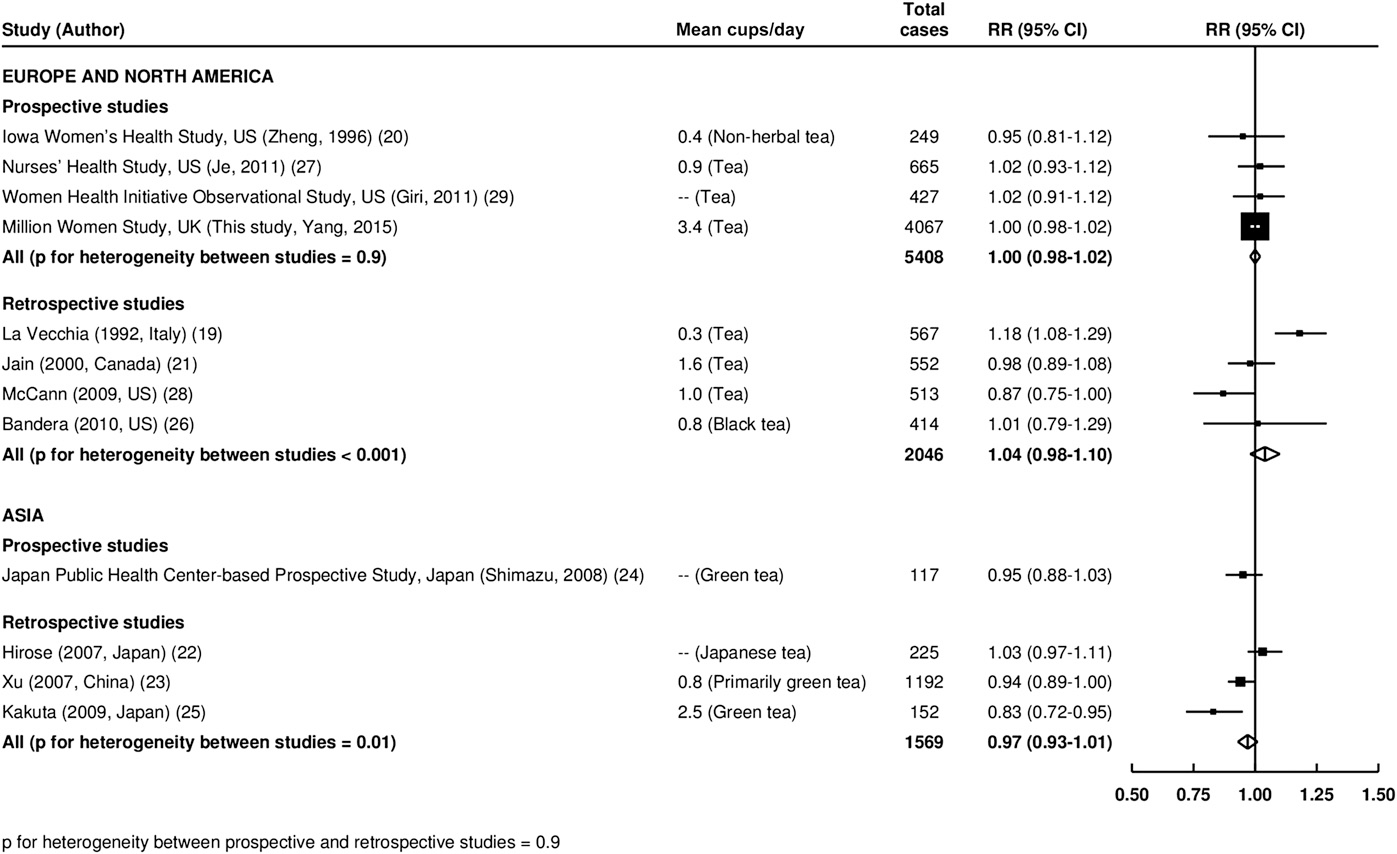
Meta-analysis of RRs (95% CIs) of endometrial cancer per additional cup of tea daily. Squares and lines represent the RR and 95% CI for each study. Square sizes are proportional to the inverses of study variances. Summary RRs and 95% CIs are shown as diamonds. Mean consumption of tea was estimated by categorical midpoints weighted by case numbers. In Hirose et al. (22) and Giri et al. (29), only RRs of the top and bottom categories were given. We assigned values of 9 and 6 cups/d for the top category of ≥7 and ≥4 cups tea, respectively. In Shimazu et al. (24), a variance-weighted least-squares method was used instead of a generalized least-squares method because case numbers by tea consumption were not available. In Bandera et al. (26) results of black tea and green tea are both provided in the article. We used the results for black tea in this analysis.

Results of the meta-analysis for risk of endometrial cancer per additional cup of tea daily are shown in **Figure 1**. In European and North American studies, we identified 4 studies with a prospective data collection and 4 studies with a retrospective data collection. No significant associations were shown in either prospective or retrospective studies between tea consumption and endometrial cancer risk (RR per additional cup daily for prospective studies: 1.00; 95% CI: 0.98, 1.02). For Asian studies, results from one prospective study did not suggest an association between tea consumption and endometrial cancer risk, nor did the 3 retrospective studies. There was no heterogeneity between prospective and retrospective studies (*P* = 0.9).

**FIGURE 2:**
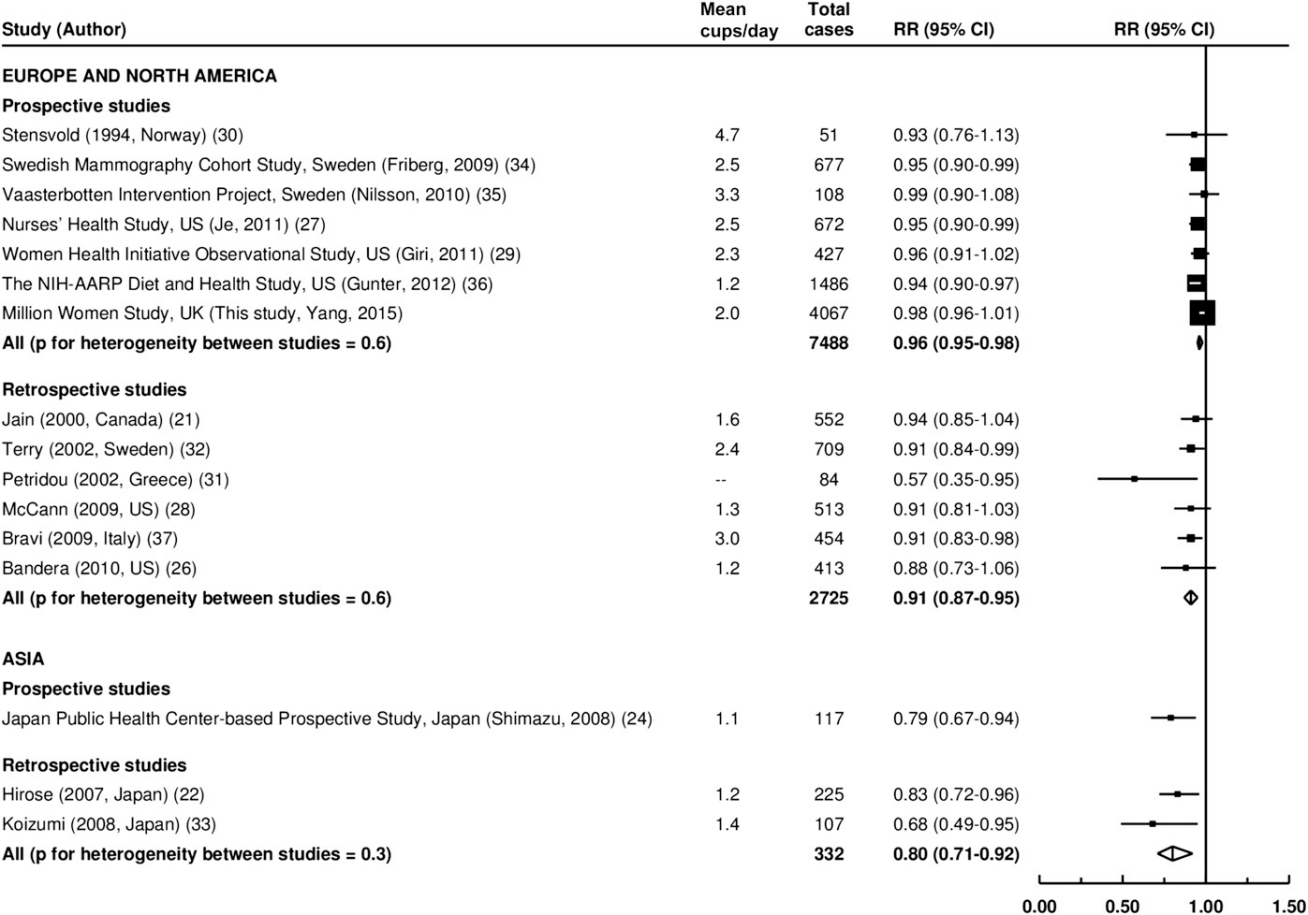
Meta-analysis of RRs of endometrial cancer per additional cup of coffee daily. Squares and lines represent the RR and 95% CI for each study. Square sizes are proportional to the inverses of study variances. Summary RRs and 95% CIs are shown as diamonds. Mean consumption of tea was estimated by categorical midpoints weighted by case numbers. In Petridou et al. (31), only RRs of drinkers compared with nondrinkers were provided. We assigned a value of 2 cups/d for drinkers in this analysis.

Results of the meta-analysis for risk of endometrial cancer per additional cup of coffee daily stratified by study design and region are shown in **Figure 2**. For European and North American studies, we included 7 prospective studies and 6 retrospective studies. There was a significantly weaker association between daily coffee intake and endometrial cancer risk in prospective than retrospective studies (RR per additional cup daily: 0.96 compared with 0.91; *P* = 0.02). For Asian studies, the numbers were small, and corresponding RRs were 0.79 (on the basis of a single study) and 0.80 (on the basis of only 2 studies). The heterogeneity between all prospective and retrospective studies was highly significant (*P* = 0.004).

## DISCUSSION

In this large cohort of United Kingdom women in middle age in whom 4067 incident endometrial cancers were registered, neither tea nor coffee intake was significantly associated with risk of endometrial cancer. Our meta-analyses showed no significant association between tea consumption and endometrial cancer risk. For coffee, findings were inconsistent and significantly heterogeneous across study designs, with prospective studies showing a weak inverse association.

Although a previous meta-analysis showed that higher consumption of tea was associated with lower risk of endometrial cancer (1), this finding was largely based on data from retrospective studies in which differential recall may have biased the results. In our prospective study, with 4067 incident endometrial cancers and a wide range of tea consumption, we showed no association between tea consumption and endometrial cancer risk either before or after multivariate adjustment or in subgroups by BMI or smoking. The addition of milk to tea was proposed to form polyphenol-protein complexes and weaken its antioxidant activity (38), but we did not show a difference in endometrial cancer risk according to whether or not milk was added. Our meta-analysis of published studies, which is an update of the most-recent meta-analysis (1), did not show a significant association between tea consumption and endometrial cancer risk in studies in Europe, North America, or Asia. Studies in Asia were mostly concerned with consumption of green tea and were relatively small.

Previous meta-analyses suggested an inverse association between coffee consumption and endometrial cancer risk (2, 3). By contrast, in our largest prospective study to date, we showed no significant association between coffee consumption and endometrial cancer risk either before or after multivariate adjustment. There was weak heterogeneity between subgroups by BMI with an inverse association between higher coffee consumption and lower endometrial cancer risk in women with BMI between 30.0 and 34.9, but this could have been a chance finding because of the multiple comparisons done. Our meta-analyses, updating results from the most recent meta-analysis (3), also suggested a possible weak inverse association between coffee consumption and endometrial cancer risk in European and North American prospective studies, an effect that was significantly smaller in prospective than in retrospective studies. There was no statistical evidence for heterogeneity across European and North American prospective studies. However, other large prospective studies for which relevant information is available have not published results for endometrial cancer. For example, the European Prospective Investigation into Cancer and Nutrition, the Netherlands Cohort Study and the Canadian National Breast Screening Study published on coffee and tea consumption in relation to ovarian cancer (39) but not endometrial cancer risk. The selective publication of only part of the prospective evidence limits the interpretation of the published evidence. In Asia, there are still too-few studies to draw reliable conclusions. Although the preparation of coffee in Asia is not likely to differ substantially from that in Europe or North America as much as the preparation of tea, RRs in our meta-analysis were significantly heterogeneous by study design and region, and more evidence is required to clarify the associations in Asian countries.

Inferences from observational studies are limited by residual confounding and measurement errors. We were able to reduce residual confounding by adjusting estimates of RRs for factors known and suspected as being associated with endometrial cancer and investigate the heterogeneity by subgroups of BMI and smoking status. Consumption of tea and coffee is usually self-reported. Although the validity or reproducibility is seldom investigated (27), we showed relatively stable intakes and good repeatability over time in this cohort for both tea and coffee consumption (5). Women in this cohort had a relatively wide range of tea and coffee consumption, which allowed sufficient case numbers in categories of extremely high consumption (≥5 cups/d) to investigate threshold effects or nonlinearity.

Caffeine has been hypothesized to affect sex hormone metabolism (40). We did not have information on whether tea or coffee consumed was decaffeinated at the time it was reported. However, on the basis of data collected in our online 24-h dietary questionnaire, which was conducted 10 y after the study baseline (41), only 6% of subjects who drank tea reported that it was nongreen and nonblack (e.g., herbal or rooibos), and only 18% of subjects who drank coffee reported that it was decaffeinated (unpublished data; *n* = 5541). These proportions were similar to what was reported in another United Kingdom cohort the UK Biobank (N Allen, UK Biobank, personal communication, 2014). Hence, our null findings for tea and coffee in relation to endometrial cancer were unlikely due to dilutions of effects because a substantial proportion of women consumed decaffeinated beverages.

In sensitivity analyses, we investigated associations after exclusion of first 4 y of follow-up, and results were not substantially different after this exclusion. Therefore, the null associations in our findings were unlikely to have been due to reverse-causation bias whereby beverage-drinking behavior may be altered by prediagnostic symptoms of endometrial cancer.

Our meta-analyses included substantially more cases than did previous ones (1–3). Studies were undertaken in Europe and North America as well as Asia, and because of differences in preparation and drinking behaviors, it was inappropriate to combine results from both regions. In these analyses, we estimated study-specific trends of endometrial cancer risk per additional cup daily. Because most studies did not provide mean consumption of tea or coffee in each nominal category, these trend estimates might have been affected by the choice of cutoffs in each study and the assumed mean consumption in each study-specific category. The trend might also have failed to reflect the true relation if the association was nonlinear. Nevertheless, we did not observe a substantial deviation from linear associations in most studies (Supplemental Tables 2 and 3), and the estimation of trends allowed almost all studies to be included and the dose response to be compared on a relative standard scale.

In conclusion, to our knowledge, this is the largest prospective study to date on the association between tea and coffee consumption and risk of endometrial cancer. Together with the findings from our meta-analyses, which added substantial new data to previous meta-analyses, our results do not provide strong support that either tea or coffee protect against endometrial cancer.

## Supplementary Material

Supplemental data
