# Supplementary material for: Tea and coffee and risk of endometrial cancer: cohort study and meta-analysis1
Source: Am J Clin Nutr. 2015 Jan 21;101(3):570–8. doi: 10.3945/ajcn.113.081836 (PMC4340062; doi:10.3945/ajcn.113.081836)
Supplement: Supplemental data [file supp_101_3_570__index.html]

Tea and coffee and risk of endometrial cancer: cohort study and meta-analysis — Tea and coffee and risk of endometrial cancer: cohort study and meta-analysis — Supplemental data 

# Tea and coffee and risk of endometrial cancer: cohort study and meta-analysis

## Supplemental data

**Files in this Data Supplement:**

- Supplemental data - Table 3
- Supplemental data - Table 1
- Supplemental data - Table 2
