# Supplementary material for: Tea and coffee and risk of endometrial cancer: cohort study and meta-analysis1
Source: Am J Clin Nutr. 2015 Jan 21;101(3):570–8. doi: 10.3945/ajcn.113.081836 (PMC4340062; doi:10.3945/ajcn.113.081836)
Supplement: Supplemental data [file 113.081836_ajcn081836SupplementaryData1.pdf]

Online Supplemental Material.

Supplementary Table 3. Studies included in Meta−analysis: Consumption of Coffee and Endometrial Cancer Risk

**Study info**	**Cases**	**Cohort info**	**Adjustment**	**Categories**	**RR by category**		**Trend (per cup/d)**	
					Unadjusted	Adjusted	Unadjusted	Adjusted
Stensvold (1994, Norway)<br>[Prospective]<br>51 cancers	51 cancers	Women aged 35−54 in 1977−1982 followed up until 1990 (10.1 years on average). Cancer cases were identified through Norwagian Cancer Registry.	Age; smoking amount; county of residence	−−	−−	−−	0.91 (0.76, 1.12)	0.93 (0.76, 1.14)
Jain (2000, Canada)<br>[Retrospective]<br>552 cancers	552 cancers	Cases were women aged 30−79 in 1994−1998; controls were from property assessment lists matched by age and area. Ontario Cancer Registry, including adenocarcinoma, carcinoma, cystadenocarcinoma, or mixed Mullerian carcinoma of the endometrium (ICD9=182)	Total energy; age; body weight; smoking; DM; OC; HT; university education; parity; age at menarche	0 g/day<br>1−250 g/day<br>251−500 g/day<br>>500 g/day	−−	1 (reference)<br>0.8 (0.54, 1.18)<br>1.18 (0.78, 1.79)<br>0.68 (0.45, 1.04)	−−	0.94 (0.85, 1.04)
Terry (2002, Sweden)<br>[Retrospective]<br>709 cancers	709 cancers	Cases were postmenopausal women aged 50−74 in 1994−1995 through national cancer registry; control were from population register.	Age; BMI; smoking; physical activity; DM; consumption of total food, fatty fish, fruit, vegetables, dairy products, high−fibre grains, legumes, and meat.	Q1: 4 time/week<br>Q2: 11 time/week<br>Q3: 22 time/week<br>Q4: 30 time/week	1 (reference)<br>0.9 (0.6, 1.2)<br>0.8 (0.6, 1)<br>0.7 (0.5, 1)	1 (reference)<br>0.9 (0.6, 1.3)<br>0.8 (0.6, 1.1)<br>0.7 (0.5, 1)	0.91 (0.85, 0.99)	0.91 (0.84, 0.99)
Petridou (2002, Greece)<br>[Retrospective]<br>84 cancers	84 cancers	Cases were women with histologically confirmed endometrial cancer in the University of Athens teaching hospital in 1999; controls were from the same hospital for small gynaecological operations, mainly pelvic prolapse, matched by age.	Age; education; height; BMI; age at menarche; menopausal status; pregnancies and abortions; alcohol; smoking; cholecystectomy	<4 cup/week<br>4+ cup/week	−−	1 (reference)<br>0.39 (0.17, 0.93)	−−	0.58 (0.35, 0.95)
Hirose (2007, Japan)<br>[Retrospective]<br>225 cancers	225 cancers	Cases were women aged 40−79 in 1990−2000; controls were from the same hospital. Histological diagnosis of endometrial cancer.	Age; year; motivation for consultation; parity; age at first birth; smoking; drinking; type of breakfast; fondness of salty and fatty foods; fruid; vegetable; beef; fish; carrot; exercise; BMI	no drinking<br>occasionally<br>1−2 cup/day<br>3+ cup/day	1 (reference)<br>0.87 (0.61, 1.26)<br>0.79 (0.57, 1.09)<br>0.54 (0.43, 0.94)	1 (reference)<br>0.7 (0.45, 1.08)<br>0.64 (0.43, 0.94)<br>0.41 (0.19, 0.87)	0.89 (0.82, 0.96)	0.83 (0.72, 0.96)
Japan Public Health Center−based Prospective Study, Japan (Shimazu 2008)<br>[Prospective]<br>117 cancers	117 cancers	Women aged 40−59 (cohort 1, 1990) or 40−69 (cohort 2, 1993) followed up until 2005. Hospital diagnosis (ICDO3=C54.0−C54.9).	Age; area; BMI; menopausal status; age at menopause; parity; exogenous female hormones; smoking status; consumption of green vegetable, beef, pork, and green tea	<=2 day/week<br>3−4 day/week<br>1−2 cup/day<br>3+ cup/dsay	1 (reference)<br>0.99 (0.57, 1.72)<br>0.64 (0.4, 1.01)<br>0.38 (0.16, 0.9)	1 (reference)<br>0.97 (0.56, 1.68)<br>0.61 (0.39, 0.97)<br>0.38 (0.16, 0.91)	0.8 (0.68, 0.94)	0.79 (0.67, 0.94)

* group−specific confidence intervals. Abbreviations: BMI body mass index; DM diabetes mellitus; HT hormonal therapy for menopause; OC oral contraceptives; RR: relative risks; WHR waist−hip ratio

**Study info**	**Cases**	**Cohort info**	**Adjustment**	**Categories**	**RR by category**		**Trend (per cup/d)**	
					Unadjusted	Adjusted	Unadjusted	Adjusted
Koizumi (2008, Japan)<br>[Retrospective]<br>107 cancers	107<br>cancers	Cases were women with Histopathologically diagnosed endometrial endometrioid adenocarcinoma, aged <80 years in 2002−2005; controls were from cancer screening programme, matched by age and area of residence	Education; BMI; smoking; age at menarche; parity; OC; DM; total calorie intake	<4 time/week<br>5−7 time/week<br>2−3 cup/day	1 (reference)<br>0.4 (0.2, 0.8)<br>0.5 (0.3, 0.8)	1 (reference)<br>0.6 (0.3, 1.2)<br>0.4 (0.2, 0.9)	0.79 (0.64, 0.97)	0.68 (0.49, 0.95)
Bravi (2009, Italy)<br>[Retrospective]<br>454 cancers	454<br>cancers	Cases were women aged 18−79 (median 60) in 1992−2006, identified using ICD9=182.0(endometrium); controls were aged 19−80 (median 61) from the same hospitals with acute non−neoplastic conditions, matched by age	Study centre; age; year of interview; education; total energy intake; BMI; diabetes; age at menarche; parity; OC; HT; menopausal status	Q1:7 cup/week<br>Q2:14 cup/week<br>Q3: 21 cup/week<br>Q4: 28 cup/week<br>Q5: NA	−−	1 (reference)<br>1.12 (0.81, 1.56)<br>0.95 (0.67, 1.35)<br>0.83 (0.54, 1.28)<br>0.5 (0.29, 0.86)	−−	0.91 (0.83, 0.98)
McCann (2009, US)<br>[Retrospective]<br>513 cancers	513<br>cancers	Women from the Patient Epidemiologic Data System (PEDS) conducted at Roswell Park Cancer Institute (RPCI) in 1982−1998. Hospital cancer diagnosis.	Age; HT; OC; education; smoking; BMI; menopausal status; coffee/tea as appropriate	0 cup/day<br>0.5 cup/day<br>1−2 cup/day<br>>2 cup/day	1 (reference)<br>0.86 (0.58, 1.29)<br>0.94 (0.69, 1.28)<br>0.72 (0.52, 1.01)	1 (reference)<br>0.77 (0.5, 1.18)<br>0.89 (0.63, 1.24)<br>0.71 (0.49, 1.03)	0.91 (0.82, 1.01)	0.91 (0.81, 1.03)
Swedish Mammography Cohort Study, Sweden (Friberg 2009)<br>[Prospective]<br>677 cancers	677<br>cancers	Women invited to a mammography screening programme in 1987−1990 (born in 1917−1948), followed from 1987−2007. Cancer identified through National Cancer Registry.	Age; BMI; smoking; education; age at menarche; age at menopause; OC; HT; parity; DM; total energy intake; tea consumption; intake of foods correlated with coffee such as buns, cookies, and cakes	<=1 cup/day<br>2−3 cup/day<br>4+ cup/day	1 (reference)<br>0.75 (0.62, 0.92)<br>0.72 (0.56, 0.93)	1 (reference)<br>0.77 (0.63, 0.94)<br>0.75 (0.57, 0.98)	0.94 (0.9, 0.98)	0.95 (0.9, 0.99)
Vaasterbotten Intervention Project, Sweden (Nilsson 2010)<br>[Prospective]<br>108 cancers	108<br>cancers	Residents aged>29 (median 50) in Vaasterbotten followed 1992−2007 (median 6 years). Cancer cases were from Regional cancer registry (ICD7=172 endometrium)	Age; BMI; smoking; education; recreational physical activity	<1 occasion/day<br>1−3 occasion/day<br>4+ occasion/day	1 (reference)<br>0.84 (0.44, 1.6)<br>0.81 (0.41, 1.62)	1 (reference)<br>0.92 (0.48, 1.76)<br>0.88 (0.44, 1.78)	0.98 (0.9, 1.07)	0.99 (0.9, 1.08)
Bandera (2010, US)<br>[Retrospective]<br>413 cancers	413<br>cancers	Women aged 21+ (mean 61.6) in 2001−2005; controls (mean age 64.3) from random digital dialing (age<65 years) and lists purchased from the Centers for Medicare and Medicais Services (age 65+ years); endometrial cancer identified from New Jersey State Cancer Registry.	Age; education; race; age at menarche; menopausal status; age at menopause; parity; OC; HT; BMI; smoking amount; addition of suger, honey, and milk	0 cup/day<br><=1 cup/day<br>>1−2 cup/day<br>>2 cup/day	−−	1 (reference)<br>1.05 (0.58, 1.89)<br>1.02 (0.56, 1.88)<br>0.69 (0.36, 1.33)	−−	0.88 (0.73, 1.06)
Women Health Initiative Observational Study, US (Giri 2011)<br>[Prospective]<br>427 cancers	427<br>cancers	Postmenopausal women aged 50−79 followed for 7.5 years on average. Cancer cases were self−reported every three years and adjudicated by physicians.	Age; ethnicity; HT type; smoking; BMI	<1 cup/day<br>1 cup/day<br>2−3 cup/day<br>4+ cup/day	1 (reference)<br>1.07 (0.8, 1.43)<br>0.92 (0.73, 1.5)<br>0.85 (0.63, 1.15)	1 (reference)<br>1.12 (0.84, 1.5)<br>0.91 (0.72, 1.16)<br>0.86 (0.63, 1.18)	0.97 (0.92, 1.02)	0.96 (0.91, 1.02)

* group−specific confidence intervals. Abbreviations: BMI body mass index; DM diabetes mellitus; HT hormonal therapy for menopause; OC oral contraceptives; RR: relative risks; WHR waist−hip ratio

Study info	Cases	Cohort info	Adjustment	Categories	RR by category		Trend (per cup/d)	
					Unadjusted	Adjusted	Unadjusted	Adjusted
Nurses' Health Study, US (Je 2011)<br>[Prospective]<br>672 cancers	672<br>cancers	Women aged 30−55 recruited in 1976 and followed from 1980 to 2006; outcomes were self−reported and ascertained with medical records (invasive endometrial adenocarcinoma).	Age, BMI, age at menopause, age at menarche, parity, age at last birth, age at last birth, duration of oral contraceptive use, postmenopausal hormone use, smoking, alcohol intake, total energy intake	<1 cup/day<br>1 cup/day<br>2−3 cup/day<br>4+ cup/day	1 (reference)<br>0.95 (0.76, 1.19)<br>0.81 (0.67, 0.98)<br>0.61 (0.47, 0.79)	1 (reference)<br>1.04 (0.83, 1.31)<br>0.93 (0.76, 1.14)<br>0.75 (0.57, 0.97)	0.91 (0.87, 0.95)	0.95 (0.9, 0.99)
The NIH−AARP Diet and Health Study, US (Gunter 2012)<br>[Prospective]<br>1486 cancers	1486<br>cancers	Women aged 50−72 in 1995−1996 were followed until 2006 (mean 9.3 years). Cancer cases from State cancer registries (ICDO3=C54.0−54.9).	Age, smoking, BMI, age at menarche, age at first childâ s birth, parity, age at menopause, HT use, oral contraceptive use, diabetes and physical activity	0 cup/day<br><1 cup/day<br>1 cup/day<br>2−3 cup/day<br>>3 cup/day	1 (reference)<br>0.89 (0.75, 1.06)<br>0.82 (0.69, 0.98)<br>0.85 (0.72, 0.99)<br>0.64 (0.52, 0.8)	1 (reference)<br>0.87 (0.73, 1.05)<br>0.82 (0.68, 0.98)<br>0.83 (0.71, 0.97)<br>0.64 (0.51, 0.8)	0.94 (0.91, 0.97)	0.94 (0.9, 0.97)
Iowa Women's Health Study, US (Uccella 2013)<br>[Prospective]<br>471 cancers	471<br>cancers	Women aged 55−69 were followed from 1986 through 2005 through via annual linkage with the Iowa Cancer Registry	Age, diabetes, duration of hormone therapy use, hypertension, age at menarche, body mass index, waist to hip ratio, smoking status and amount, total energy, alcohol use	Never or <= once per month<br>< 1 cup per week<br><1 cup per day<br>2−3 cups per day<br>4+ cups per day	1<br>0.89<br>0.65<br>0.8<br>0.54	1 (reference)<br>0.95 (0.66, 1.36)<br>0.75 (0.52, 1.09)<br>0.95 (0.71, 1.28)<br>0.71 (0.51, 0.99)	−−	−−
Million Women Study, UK (This study, Yang 2015)<br>[Prospective]<br>4067 cancers	4067<br>cancers	Women in middle age recruited in 1996−2001 and followed from 1999−2005 through 2011 by linking to National Cancer Registry.	Age, region, neighbourhood social deprivation, height, age at menarche, parity, duration of oral contraceptive use, age and status of menopause at study baseline, duration of hormone therapy for menopause, body mass index, smoking, alcohol consumption, strenuous exercise, tea consumption, and other non−alcohol fluid intake	<1 cup/day<br>1−2 cups/day<br>3−4 cups/day<br>5+ cups/day	0.99 (0.93, 1.06)<br>1 (0.95, 1.05)<br>0.97 (0.9, 1.04)<br>0.96 (0.87, 1.06)	0.99 (0.92, 1.06)<br>1 (0.95, 1.05)<br>0.94 (0.88, 1.01)<br>0.92 (0.82, 1.03)	0.99 (0.97, 1.01)	0.98 (0.96, 1.01)

* group−specific confidence intervals. Abbreviations: BMI body mass index; DM diabetes mellitus; HT hormonal therapy for menopause; OC oral contraceptives; RR: relative risks; WHR waist−hip ratio
