# Supplementary material for: Tea and coffee and risk of endometrial cancer: cohort study and meta-analysis1
Source: Am J Clin Nutr. 2015 Jan 21;101(3):570–8. doi: 10.3945/ajcn.113.081836 (PMC4340062; doi:10.3945/ajcn.113.081836)
Supplement: Supplemental data [file 113.081836_ajcn081836SupplementaryData2.pdf]

Online Supplemental Material.

## Supplementary Table 1: Relative risks (RR) and confidence intervals or group-specific confidence intervals† (CIs) for associations of endometrial cancer risk with tea or coffee consumption: sensitivity analyses

### Analysis 1: Excluding first 4 years of follow up

| | | Tea consumption (cups/day) | | | | p for heterogeneity | Trend (per cup) | |
|---|---|---|---|---|---|---|---|---|
| | | **<1** | **1-2** | **3-4** | **5+** | | All | daily consumers only |
| **No exclusion #** | | | | | | | | |
| | Cases | 647 | 893 | 1374 | 1153 | | 4067 | 3420 |
| | $RR_a$ (95% CI) | 1.04 (0.96, 1.14) | 1.00 (0.94, 1.07) | 1.05 (1.00, 1.11) | 1.01 (0.95, 1.08) | 0.6 | 1.00 (0.98, 1.02) | 1.00 (0.97, 1.02) |
| **Excluding first 4 years of follow up #** | | | | | | | | |
| | Cases | 415 | 549 | 863 | 739 | | 2566 | 2151 |
| | $RR_a$ (95% CI) | 1.09 (0.98, 1.22) | 1.00 (0.92, 1.09) | 1.08 (1.01, 1.16) | 1.07 (0.98, 1.16) | 0.4 | 1.00 (0.98, 1.03) | 1.01 (0.98, 1.04) |

| | | Coffee consumption (cups/day) | | | | p for heterogeneity | Trend (per cup) | |
|---|---|---|---|---|---|---|---|---|
| | | **<1** | **1-2** | **3-4** | **5+** | | All | daily consumers only |
| **No exclusion #** | | | | | | | | |
| | Cases | 1009 | 1839 | 842 | 377 | | 4067 | 3058 |
| | $RR_a$ (95% CI) | 0.99 (0.92, 1.06) | 1.00 (0.95, 1.05) | 0.94 (0.88, 1.01) | 0.92 (0.82, 1.03) | 0.4 | 0.98 (0.96, 1.01) | 0.97 (0.94, 1.01) |
| **Excluding first 4 years of follow up #** | | | | | | | | |
| | Cases | 620 | 1165 | 545 | 236 | | 2566 | 1946 |
| | $RR_a$ (95% CI) | 0.95 (0.87, 1.04) | 1.00 (0.94, 1.06) | 0.95 (0.88, 1.03) | 0.88 (0.76, 1.01) | 0.4 | 0.98 (0.95, 1.02) | 0.97 (0.93, 1.01) |

### Analysis 2: Additional adjustment for daily sugar added in cereal, tea, coffee, or fruit etc. (n=480,513)

| | | Tea consumption (cups/day) | | | | p for heterogeneity | Trend (per cup) | |
|---|---|---|---|---|---|---|---|---|
| | | **<1** | **1-2** | **3-4** | **5+** | | All | daily consumers only |
| **Without additional adjustment #** | Cases | 554 | 769 | 1172 | 994 | | 3489 | 2935 |
| | $RR_a$ (95% CI) | 1.05 (0.95, 1.15) | 1.00 (0.93, 1.07) | 1.05 (0.99, 1.11) | 1.00 (0.93, 1.07) | 0.6 | 0.99 (0.97, 1.01) | 0.99 (0.97, 1.02) |
| **Additional adjustment for daily sugar added in cereal, tea, coffee, or fruit etc. ‡** | $RR_a$ (95% CI) | 1.05 (0.96, 1.15) | 1.00 (0.93, 1.07) | 1.05 (0.99, 1.11) | 1.00 (0.93, 1.07) | 0.6 | 0.99 (0.97, 1.01) | 0.99 (0.97, 1.02) |

| | | Coffee consumption (cups/day) | | | | p for heterogeneity | Trend (per cup) | |
|---|---|---|---|---|---|---|---|---|
| | | **<1** | **1-2** | **3-4** | **5+** | | All | daily consumers only |
| **Without additional adjustment #** | Cases | 868 | 1585 | 707 | 329 | | 3489 | 2621 |
| | $RR_a$ (95% CI) | 1.00 (0.93, 1.08) | 1.00 (0.95, 1.05) | 0.92 (0.86, 0.99) | 0.93 (0.83, 1.05) | 0.3 | 0.98 (0.95, 1.01) | 0.97 (0.94, 1.01) |
| **Additional adjustment for daily sugar added in cereal, tea, coffee, or fruit etc. ‡** | $RR_a$ (95% CI) | 1.00 (0.93, 1.08) | 1.00 (0.95, 1.05) | 0.93 (0.86, 1.00) | 0.94 (0.83, 1.05) | 0.4 | 0.98 (0.95, 1.01) | 0.98 (0.94, 1.01) |

#Age as underlying variable. Risks were adjusted for region, socioeconomic status, height, age at menarche, parity, duration of oral contraceptive use, age and status of menopause at study baseline, duration of hormone therapy for menopause, body mass index, smoking, alcohol consumption, strenuous exercise, tea or coffee consumption (as appropriate), and other non-alcohol fluid intake
† group-specific confidence intervals were used to allow comparison of risks between any two groups, even when neither is the reference group.
‡ <1, 1-2, 3 or more spoons of sugar added daily
