# Supplementary material for: Tea and coffee and risk of endometrial cancer: cohort study and meta-analysis1
Source: Am J Clin Nutr. 2015 Jan 21;101(3):570–8. doi: 10.3945/ajcn.113.081836 (PMC4340062; doi:10.3945/ajcn.113.081836)
Supplement: Supplemental data [file 113.081836_ajcn081836SupplementaryData3.pdf]

Online Supplemental Material.

Supplementary Table 2. Studies included in Meta−analysis: Consumption of Tea and Endometrial Cancer Risk

Study info	Cases	Cohort info	Adjustment	Categories	RR by category		Trend (per cup/d)	
					Unadjusted	Adjusted	Unadjusted	Adjusted
La Vecchia (1992, Italy)<br>[Retrospective]<br>567 cancers	567 cancers	Cases were women aged <75 years in 1983−1990; controls were patients for non−neoplastic conditions. Histologically confirmed cancer of the endometrium.	Age; area of residence; education; smoking; coffee consumption	nonusers<br>1+ cup/day	−−	1 (reference)<br>1.4 (1.2, 1.7)	−−	1.18 (1.08, 1.29)
Jain (2000, Canada)<br>[Retrospective]<br>552 cancers	552 cancers	Cases were women aged 30−79 in 1994−1998; controls were from property assessment lists matched by age and area. Ontario Cancer Registry, including adenocarcinoma, carcinoma, cystadenocarcinoma, or mixed Mullerian carcinoma of the endometrium (ICD9=182)	Total energy; age; body weight; smoking; DM; OC; HT; university education; parity; age at menarche	0 g/day<br>1−250 g/day<br>251−500 g/day<br>>500 g/day	−−	1 (reference)<br>1.21 (0.87, 1.68)<br>1.17 (0.79, 1.73)<br>0.99 (0.68, 1.45)	−−	0.98 (0.89, 1.08)
Xu (2007, China)<br>[Retrospective]<br>1192 cancers	1192 cancers	Cases were women aged 30−69 in 1997−2003; controls were from resident registry matched by age in 1996. Cancer cases from Shanghai Cancer Registry.	Age; education; menopausal status; years of menstruation; parity; BMI; alcohol consumption; BMI; physical activity; energy intake; total fruit and vegetable intake; soy protein intake	Never<br><=1500 g/year<br>1500+ g /year	−−	1 (reference)<br>0.8 (0.6, 1)<br>0.8 (0.6, 1)	−−	0.94 (0.89, 1)
Hirose (2007, Japan)<br>[Retrospective]<br>225 cancers	225 cancers	Cases were women aged 40−79 in 1990−2000; controls were from the same hospital. Histological diagnosis of endometrial cancer.	Age; year; motivation for consultation; parity; age at first birth; smoking; drinking; type of breakfast; fondness of salty and fatty foods; fruid; vegetable; beef; fish; carrot; exercise; BMI	Occasional or non−drinkers<br>7+ cup/day	−−	1 (reference)<br>1.33 (0.75, 2.35)	−−	1.03 (0.97, 1.11)
Japan Public Health Center−based Prospective Study, Japan (Shimazu 2008)<br>[Prospective]<br>117 cancers	117 cancers	Women aged 40−59 (cohort 1, 1990) or 40−69 (cohort 2, 1993) followed up until 2005. Hospital diagnosis (ICDO3=C54.0−C54.9).	Age; area; BMI; menopausal status; age at menopause; parity; exogenous female hormones; smoking status; consumption of green vegetable, beef, pork, and green tea	<1 cup/day<br>1−2 cup/day<br>3−4 cup/day<br>5+ cup/day	−−	1 (reference)<br>1.04 (0.62, 1.74)<br>0.79 (0.47, 1.35)<br>0.75 (0.44, 1.3)	−−	0.95 (0.88, 1.03)
McCann (2009, US)<br>[Retrospective]<br>513 cancers	513 cancers	Women from the Patient Epidemiologic Data System (PEDS) conducted at Roswell Park Cancer Institute (RPCI) in 1982−1998. Hospital cancer diagnosis.	Age; HT; OC; education; smoking; BMI; menopausal status; coffee/tea as appropriate	0 cup/day<br>0.5 cup/day<br>1−2 cup/day<br>>2 cup/day	1 (reference)<br>0.8 (0.58, 1.09)<br>0.95 (0.69, 1.31)<br>0.59 (0.38, 0.91)	1 (reference)<br>0.81 (0.57, 1.14)<br>0.89 (0.63, 1.26)<br>0.56 (0.35, 0.9)	0.89 (0.78, 1.02)	0.87 (0.75, 1)

* group−specific confidence intervals. Abbreviations: BMI body mass index; DM diabetes mellitus; HT hormonal therapy for menopause; OC oral contraceptives; RR: relative risks; WHR waist−hip ratio

**Study info**	**Cases**	**Cohort info**	**Adjustment**	**Categories**	**RR by category**		**Trend (per cup/d)**	
					Unadjusted	Adjusted	Unadjusted	Adjusted
Kakuta (2009, Japan)<br>[Retrospective]<br>152 cancers	152<br>cancers	Cases were women aged <80 years in 2002−2007; controls were from cancer screening programme, matched by age and area of residence. Histopathologically diagnosed endometrial endometrioid adenocarcinoma	BMI; education; parity; menopausal status; smoking; DM; total calorie intake; consumption of miso soup, tofu, or coffee	<4 cup/week<br>5−7 cup/week<br>2−3 cup/day<br>4+ cup/day	1 (reference)<br>0.85 (0.49, 1.45)<br>0.6 (0.35, 1.03)<br>0.51 (0.28, 0.92)	1 (reference)<br>0.77 (0.37, 0.58)<br>0.61 (0.3, 1.23)<br>0.33 (0.15, 0.75)	0.89 (0.81, 0.98)	0.83 (0.72, 0.95)
Bandera (2010, US)<br>[Retrospective]<br>414 cancers	414<br>cancers	Women aged 21+ (mean 61.6) in 2001−2005; controls (mean age 64.3) from random digital dialing (age<65 years) and lists purchased from the Centers for Medicare and Medicais Services (age 65+ years); endometrial cancer identified from New Jersey State Cancer Registry.	Age; education; race; age at menarche; menopausal status; age at menopause; parity; OC; HT; BMI; smoking amount; addition of suger, honey, and milk	0 cup/day<br><=1 cup/day<br>>1 cup/day	−−	1 (reference)<br>2.24 (1.29, 3.88)<br>1.77 (0.96, 3.28)	−−	1.01 (0.79, 1.29)
Women Health Initiative Observational Study, US (Giri 2011)<br>[Prospective]<br>427 cancers	427<br>cancers	Postmenopausal women aged 50−79 followed for 7.5 years on average. Cancer cases were self−reported every three years and adjudicated by physicians.	Age; ethnicity; HT type; smoking; BMI; coffee consumption	<1 cup/day<br>4+ cup/day	−−	1 (reference)<br>1.1 (0.61, 1.97)	−−	1.02 (0.91, 1.13)
Nurses' Health Study, US (Je 2011)<br>[Prospective]<br>665 cancers	665<br>cancers	Women aged 30−55 recruited in 1976 and followed from 1980 to 2006; outcomes were self−reported and ascertained with medical records (invasive endometrial adenocarcinoma).	Age, BMI, age at menopause, age at menarche, parity, age at last birth, age at last birth, duration of oral contraceptive use, postmenopausal hormone use, smoking, alcohol intake, total energy intake, total coffee intake	<1 cup/mth<br>1 cup/mth−/day<br>1 cup/day<br>2+ cup/day	1 (reference)<br>1.18 (0.94, 1.49)<br>1.39 (1.06, 1.84)<br>1.2 (0.88, 1.63)	1 (reference)<br>1.1 (0.87, 1.4)<br>1.24 (0.94, 1.65)<br>1.06 (0.77, 1.46)	1.06 (0.97, 1.16)	1.02 (0.93, 1.12)
Iowa Women's Health Study, US (Uccella 2013)<br>[Prospective]<br>471 cancers	471<br>cancers	Women aged 55−69 were followed from 1986 through 2005 through via annual linkage with the Iowa Cancer Registry	Age, diabetes, duration of hormone therapy use, hypertension, age at menarche, body mass index, waist to hip ratio, smoking status and amount, total energy, alcohol use	Never or <= once per month<br>1−3 cup per mont<br>1−4 cups per week<br>5+ cups a week	1<br>0.87<br>0.99<br>1.05	1 (reference)<br>0.87 (0.66, 1.15)<br>0.89 (0.69, 1.15)<br>0.95 (0.74, 1.22)	−−	−−
Million Women Study, UK (This study, Yang 2015)<br>[Prospective]<br>4067 cancers	4067<br>cancers	Women in middle age recruited in 1996−2001 and followed from 1999−2005 through 2011 by linking to National Cancer Registry.	Age, region, neighbourhood social deprivation, height, age at menarche, parity, duration of oral contraceptive use, age and status of menopause at study baseline, duration of hormone therapy for menopause, body mass index, smoking, alcohol consumption, strenuous exercise, coffee consumption, and other non−alcohol fluid intake	<1 cup/day<br>1−2 cups/day<br>3−4 cups/day<br>5+ cups/day	1.11 (1.02, 1.2)<br>1 (0.94, 1.07)<br>1.07 (1.01, 1.13)<br>1.04 (0.98, 1.11)	1.04 (0.96, 1.14)<br>1 (0.94, 1.07)<br>1.05 (1, 1.11)<br>1.01 (0.95, 1.08)	1 (0.98, 1.01)	1 (0.98, 1.02)

* group−specific confidence intervals. Abbreviations: BMI body mass index; DM diabetes mellitus; HT hormonal therapy for menopause; OC oral contraceptives; RR: relative risks; WHR waist−hip ratio
